# *Clostridium difficile* cure with fecal microbiota transplantation in a child with Pompe disease: a case report

**DOI:** 10.1186/s13256-018-1659-2

**Published:** 2018-04-28

**Authors:** D. E. Dow, P. C. Seed

**Affiliations:** 1 Division of Infectious Diseases, Department of Pediatrics, Duke University Medical Center, Box 3499, Durham, NC 27710 USA; 2 Division of Infectious Diseases, Department of Pediatrics, Feinberg School of Medicine, Northwestern University, Evanston, USA

**Keywords:** Refractory *Clostridium difficile*, Fecal microbiota transplantation, Medically complex children

## Abstract

**Background:**

Recurrent *Clostridium difficile* infection is a growing problem among children due to both the increasing survival of medically fragile children with complicated chronic medical conditions resulting in prolonged antibiotic exposure and hospitalization and the emergence of strains of *Clostridium difficile* that are hypervirulent and associated with high rates of relapse.

**Case presentation:**

This case describes a medically complex 21-month-old Hispanic girl with Pompe disease and B cell immunodeficiency with recurrent *Clostridium difficile* infection refractory to antimicrobial management. She presented with nine recurrent episodes of *Clostridium difficile* infection including fever, foul smelling diarrhea, and respiratory distress with failed sustained responses to compliant treatment using metronidazole and pulsed vancomycin therapy. Maternal donor fecal microbiota transplantation was performed with complete symptom resolution and produced a sustained cure, now 5 years in duration.

**Conclusions:**

This patient presented with symptomatic *Clostridium difficile* at an early age causing significant morbidity and reduced quality of life. After nearly one year of failed medical management, fecal microbiota transplantation provided a cure. Further evidence-based research is necessary to test the safety and efficacy of this low technology, low cost, and morbidity-sparing therapy in children.

## Background

*Clostridium difficile* infection (CDI) has been steadily increasing among children over the last decade [1–4], resulting in increased hospitalizations, morbidity, and cost [5, 6]. The factors that account for this increasing incidence are not entirely understood, but include the emergence of the hypervirulent and multidrug-resistant *C. difficile* strain, NAP1/BI/027 [7, 8], the strain DH/NAP11/106 [9] which is associated with high rates of relapse, and the increasing survival of children with complicated chronic medical conditions that result in increased antibiotic exposure and prolonged hospitalization.

*C. difficile* is a Gram-positive, anaerobic, spore-forming bacterium. It is acquired through the fecal–oral route, traditionally during exposure to the health care environment, though it is increasingly recognized to be acquired in the community [1, 2, 10]. *C. difficile* persists in the environment and on home and hospital surfaces for months to years and resists many methods of elimination [11]. The pathogenesis of CDI begins with disruption of the normally diverse intestinal microbiota, which establishes nutritional niches and metabolic pathways that prevent colonization of *C. difficile* and other pathogens in a healthy host. Antibiotics can alter this homeostatic environment leading to colonic dysbiosis that predisposes the host to pathogenic bacteria, such as *C. difficile*. Sporulation and germination of *C. difficile* leads to exotoxin A (TcdA) and exotoxin B (TcdB) production that have cytotoxic and proinflammatory effects on the gastrointestinal epithelium [12]. Without an adequate immune response, CDI symptoms may include diarrhea, leukocytosis, fever, abdominal pain, and pseudomembranous colitis, and can progress to toxic megacolon requiring abdominal surgery such as colectomy, septic shock, and even death [6, 13].

Established risk factors for CDI include antibiotic exposure, prolonged hospitalization, bowel manipulation, or surgery including gastrointestinal feeding devices and immunosuppression [6, 8, 14]. Not all antibiotics pose the same risk for CDI; clindamycin and cephalosporins, along with fluoroquinolones and penicillins pose the greatest risk, presumably producing specific disruptions in microbiota homeostasis that open new niches for *C. difficile* to flourish [15]. The medical necessity for recurrent antibiotic exposure, feeding devices, and hospitalizations among medically complex children create a persistent risk state for recurrent CDI once infection is established.

Treatment guidelines and a pediatric-specific policy statement are published for CDI [15, 16]. Goals of therapy are shared by both publications and include discontinuation of inciting antibiotic therapy, or narrowing the antibiotic spectrum if antibiotics are necessary to treat an underlying condition. Metronidazole remains the drug of choice for initial mild infections; orally administered vancomycin, although more expensive and increasing the risk of vancomycin-resistant *Enterococcus* species infection, is indicated for severe infections, even if they are the first occurrence [17]. CDI recurrence is generally defined as new CDI within 8 weeks, or up to 12 weeks for children, after onset and resolution of the previous episode [15, 18]. Data suggest once there is a first recurrence, patients are 35–40% more likely to have a second recurrence, [19, 20] and have a 60% risk after a second recurrent CDI [21, 22]. In children, 30% have recurrence of infection either from the same strain, or exposure to a new strain [8]. The first relapse is treated with the same drug as initial infection depending on disease severity; use of metronidazole is not recommended beyond two courses due to potential neurotoxicity. For multiple recurrences, tapered/pulsed orally administered vancomycin, rifaximin, nitazoxanide, or fidaxomicin have been used but without specific evidence to support the practice [8]. Probiotics such as *Saccharomyces boulardii* have modest utility [15]. In refractory cases, fecal microbiota transplantation (FMT) involving instillation of stool from a healthy donor to restore the indigenous enterome has provided higher rates of cure than traditional antibiotic therapy [19].

## Case presentation

A 21-month-old Hispanic girl born full term via cesarean was diagnosed as having cross-reactive immunologic material negative (CRIM-negative) Pompe disease at 4 months of age. Her disease was complicated by dilated cardiomyopathy, ventilator dependence, and failure to thrive requiring primary nutrition by a gastrojejunostomy (GJ) feeding tube. At 6 months of life, she initiated alglucosidase alfa therapy and treatment to limit immune rejection of the enzyme therapy with rituximab and methotrexate, eliciting a B cell immunodeficient state. At 9 months of life, she presented with recurrent foul-smelling diarrhea, fever, and abdominal discomfort while receiving broad-spectrum antibiotics (piperacillin/tazobactam) for presumed aspiration pneumonia based on her initial symptoms of cough and fever. The diagnosis of CDI was confirmed by polymerase chain reaction (PCR) for *C. difficile* toxin and exclusion of other infectious etiology. Her CDI was treated with metronidazole with symptom resolution and subsequent negative PCR for toxin; however, her symptoms and positive PCR continued to recur soon after cessation of antibiotic therapy (Table 1). With each new recurrence, she presented with fever, diarrhea, and decompensated with respiratory distress, hospital admission, and use of systemic antibiotics; a recurrent cycle leading to significant morbidity and reduced quality of life.

**Table 1:** Timeline of recurrent *Clostridium difficile* infection and treatment strategies

Recurrence	Confirmatory positive *C. difficile* PCR	Treatment
	February 10, 2010	Metronidazole × 7 days
1	March 10, 2010	Metronidazole × 7 days
2	April 8, 2010	Metronidazole × 10 days
3	May 20, 2010	Metronidazole × 10 days
4	June 7, 2010	Orally administered vancomycin × 10 days
5	July 3, 2010	Orally administered vancomycin × 6 weeks with taper
6	November 10, 2010	Orally administered vancomycin × 6 weeks with taper
7	December 2, 2010	
8	December 31, 2010	approached for fecal microbial transplant
9	February 21, 2011	Fecal microbiota transplant February 24, 2011
None	All subsequent PCR negative to date (last PCR sent April 2, 2016)	

*PCR* polymerase chain reaction

After 12-months of recurrent CDI, four courses of metronidazole, and one short course of vancomycin followed by two rounds of tapered vancomycin therapy, she continued to experience symptoms of fever, foul smelling diarrhea, and respiratory distress. FMT was presented as an alternative therapy. The child’s biological mother, originally from El Salvador, was screened to be the fecal donor using a previously described protocol [23]. Her mother, a previously healthy woman of normal body habitus, had no history of parasitic infections, typhoid fever, or dysentery, no recent antibiotic use, no immunosuppressant drug use, no metabolic disorders, and no history of colonic polyps or cancers. Laboratory testing included screening for human immunodeficiency virus (HIV), hepatitis A, hepatitis B, hepatitis C, rapid plasma reagin (RPR), and *Trypanosoma cruzi* serologies which were all negative. A complete blood count and complete metabolic profile were within normal limits. Two separate stool samples were screened: by culture for enteric pathogens including *Giardia*, *Cryptosporidium*, and *Yersinia*; for *C. difficile* toxin by PCR; and for ova and parasites at 3 weeks and 1 week prior to transplant. All studies were negative.

Five days prior to transplant our patient’s symptoms reappeared, and vancomycin was initiated (40 mg/kg per day divided each 6 hours), her parents agreed to a signed consent for fecal transplantation. On the day of transplant, our patient was afebrile, her vital signs were stable with weight of 12 kg on respiratory ventilation, and her physical examination was within normal limits for her baseline. The freshly donated maternal stool sample weighed 11 g and was homogenized with 100 mL normal saline. The stool was then filtered through sterile gauze and a coffee filter to remove all significant particulate matter. Our patient’s longstanding GJ tube was accessed and flushed with 15 ml of saline through the jejunal inlet; 35 ml of the stool filtrate was then administered, followed by 15 ml normal saline flush without complication, all within approximately 4 hours of the sample donation. Her vital signs were measured prior to the transplant and every 30 minutes for 2 hours after the transplant. She was discharged home with instructions to resume regular feeds and medications. Orally administered vancomycin was discontinued. To date (5-years post-fecal transplant), our patient remains PCR negative for *C. difficile* toxin and free of CDI symptoms. She experienced no adverse events attributable to FMT.

## Discussion

FMT has been only rarely utilized and reported among children with recurrent CDI. This case is unusual due to this child’s underlying genetic disease, immunodeficiency, and 5-year follow-up documenting a durable medical cure. This patient had several risk factors for CDI including immunocompromised state, frequent ongoing exposure to the health care environment, administration of broad-spectrum antibiotics, feeding via GJ feeding tube, and cesarean delivery. The continuous cycle of refractory CDI resulted in repeat hospital admissions and the use of broad-spectrum antibiotics, which led to treatment with FMT. The goal of FMT was to correct the underlying colonic dysbiosis and to reestablish the diverse fecal flora that acts as a host defense against *C. difficile* [19, 24, 25]. The availability of the maternal donor to restore the fecal microbial homeostasis of her child was ideal.

Controversy exists over when a child may develop CDI. Infants under 12 months have some of the highest known colonization rates, up to 50% depending on duration of hospitalization and breast versus formula feeding [26–29]. Prior research posited that infants lack the molecular machinery or toxin receptors to process *C. difficile* toxin [30]. Infants may not experience true symptomatic disease which occurs when exotoxin is produced and internalized in intestinal epithelial cells causing inflammation and cell death [16, 31, 32]. More recent work suggested that the transition from colonization to disease may reside in the immune response to *C. difficile* [33]. This patient was immunosuppressed while receiving rituximab and methotrexate and presented with symptoms suggestive of recurrent CDI.

Early, small cohort, prospective, randomized controlled trials of FMT established up to 90% efficacy and safety among adults and superiority to orally administered vancomycin [19, 21, 34, 35]; however, to date, no randomized controlled trials among children have been published. FMT using more patient and family accepted methods of delivery have led to development and testing of mass-produced, orally ingested capsules [21, 34]. Given the significant economic burden of recurrent CDI [36], FMT offers a potentially cost-effective and efficacious therapy to cure recurrent CDI in children as demonstrated in this report.

The limitations of this single case report include the young age at which this patient became symptomatic, the lack of strain typing for the hypervirulent NAP1/BI/027 and the strain DH/NAP11/106 which is associated with high rates of relapse, and lack of clonal analysis. We do not have microbiome level data from our patient or from her mother to demonstrate the pre-FMT or post-FMT community composition; however, symptom resolution and disappearance of toxin suggest the FMT did successfully supplant the *C. difficile-*associated microbiota with a more homeostatic state resistant to recurrent CDI.

## Conclusions

This report demonstrated that FMT may be safely and effectively employed in a medically complex child, although the risk–benefit relationship of recurrent CDI and FMT complications must be currently weighed on an individual basis. Evidence-based, randomized controlled trials are urgently needed to evaluate the safety and efficacy of this procedure in children.

## Abbreviations

CDI: *Clostridium difficile* infection
FMT: Fecal microbiota transplantation
GJ: Gastrojejunostomy
PCR: Polymerase chain reaction
